# Compassion fatigue in emergency care professionals

**DOI:** 10.1590/0034-7167-2023-0367

**Published:** 2025-06-20

**Authors:** Letícia de Lima Trindade, Maiara Daís Schoeninger, Josiane Carneiro, Elisabete Maria das Neves Borges, Clarissa Bohrer da Silva, Carine Vendruscolo, Fernanda Karla Metelski

**Affiliations:** I Universidade do Estado de Santa Catarina. Chapecó, Santa Catarina, Brazil; II Universidade Comunitária da região de Chapecó. Chapecó, Santa Catarina, Brazil; III Escola Superior de Enfermagem do Porto. Porto, Portugal

**Keywords:** Compassion Fatigue, Emergency Medical Services, Health Personnel, Occupational Health, Occupational Risks., Desgaste por Empatía, Servicios Médicos de Urgencia, Personal de Salud, Salud Laboral, Riesgos Laborales.

## Abstract

**Objectives:**

to assess the level of compassion fatigue in healthcare professionals working in emergency services and its association with socio-professional characteristics.

**Methods:**

cross-sectional research, carried out in 2022 and 2023, with 186 healthcare workers. A socio-occupational questionnaire and the Professional Quality of Life Scale 5 were used.

**Results:**

higher averages of satisfaction with compassion were evidenced, followed by burnout syndrome and Secondary Traumatic Stress. Age and number of children were related to satisfaction with compassion. These variables and years of experience in the health field were inversely correlated with burnout scores. Furthermore, the higher the level of education, the lower the scores for Secondary Traumatic Stress.

**Conclusions:**

compassion satisfaction provides protection for most study participants, and it is important to invest in it to avoid compassion fatigue in this scenario.

## RESUMEN

**Objetivos:**

evaluar el nivel de fatiga por compasión en profesionales de la salud que actúan en servicios de urgencia y emergencia y su asociación con características socioprofesionales.

**Métodos:**

investigación transversal, realizada en 2022 y 2023, con 186 trabajadores de la salud. Se utilizó un cuestionario sociolaboral y la *Professional Quality of Life Scale* 5.

**Resultados:**

se evidenciaron mayores promedios de satisfacción por la compasión, seguida del síndrome de burnout y del estrés traumático secundario. La edad y el número de hijos se relacionaron con la satisfacción por compasión. Estas variables y los años de experiencia en el campo de la salud se correlacionaron inversamente con las puntuaciones de agotamiento. Además, cuanto mayor es el nivel educativo, menores son las puntuaciones de Estrés Traumático Secundario.

**Conclusiones:**

la satisfacción por compasión brinda protección a la mayoría de los participantes del estudio y es importante invertir en ella para evitar la fatiga por compasión en este escenario.

## INTRODUCTION

Healthcare services are considered places that pose several threats to workers’ health. Among these threats, exposure to psychosocial risks related to the design, organization and management of work stands out^(1)^. The complexity of the healthcare field and the challenges of providing quality care place professionals at a high level of stress, which favors, among other problems, compassion fatigue (CF)^(2)^. This fatigue is characterized by behaviors and emotions resulting from the process of caring for or wanting to help a traumatized or distress person, which leads the person providing care/help to a state of biological, psychological and social exhaustion^(3)^. In its theoretical origin, CF is based on the professional quality of life (PQoL) model, which presents it as a result of the combination of burnout syndrome (BO), secondary traumatic stress (STS) and reduced compassion satisfaction (CS)^(3)^. In this regard, it is worth highlighting that BO and STS are not synonymous with CF, but their sum influences its development.

PQoL can be understood as the quality that professionals feel in relation to their work^(3,4)^. The model incorporates two aspects: positive (CS) and negative (CF). The first refers to the pleasure that workers feel when performing their job well, and is related to the fact of being able to help others, to the positive feeling towards their colleagues and to the ability to contribute to the work environment or even to the greater good of society. On the other hand, the second is divided into two dimensions: BO, a process of chronic work stress, which involves the triad of emotional exhaustion, depersonalization and low personal accomplishment; and STS, motivated by fear and work-related trauma^(4)^.

Among these aspects, BO can be defined as an occupational phenomenon caused by high levels of stress, experienced over the long term and managed unsuccessfully, causing a state of emotional exhaustion, depersonalization and feelings of incompetence^(5)^. In turn, STS is characterized as an occupational syndrome arising from stress, caused by exposure to incidents experienced by another person^(3)^. CS is conceptualized as the gratification provided by the act of caring for people in situations of distress. When professionals working in healthcare services feel valued and are satisfied with their interpersonal and work relationships, CS acts as a protective factor against CF^(3,6)^.

A study showed high rates of CF, which affect 52.55% of professionals working in healthcare services. Moreover, BO totaled 51.98%, and CS, 47.55%, respectively^(7)^. An integrative review, including more than 28,000 nurses from 11 countries, showed that the levels of the phenomenon have gradually increased over the last ten years, which raises an alert to the seriousness of the problem among healthcare professionals as well as the need for strategies and interventions that provide improvements in the sector^(8)^. A second systematic review, which included 71 articles, showed that the phenomenon exists in several groups and specialties, and can be successfully measured using the Professional Quality of Life (ProQOL) tool^(9)^.

Among the most cited consequences in literature, CF causes a reduction in professionals’ capacity and interest to act empathetically when faced with situations of distress in others, which is considered the “cost of caring”^(10)^, which can impact professional performance and put patient safety at risk^(11)^. In this direction, studies on CF have contributions to worker health, promotion of a culture of safety in institutions as well as culture of peace.

Furthermore, the consequences left by the pandemic caused by the coronavirus disease (COVID-19) intensified the stressors, which directly impacted workers’ physical and mental health^(11,12)^. As these professionals experience a new set of circumstances caused by STS, the occurrence of CF increases and job satisfaction decreases^(11)^.

Among the professionals most affected, those working in emergency services stand out, as they face stressful clinical situations on a daily basis, including events of violence, accidents, traumas and deaths^(13)^. Furthermore, these same professionals are the ones who sometimes have to deal with and convey bad news to companions and family members, generating a new stress factor^(13)^, making them vulnerable to CF.

It is worth noting that CF is a phenomenon that requires further investigation and can be considered one of the main threats to professionals’ mental health. It has an impact on the quality of care provided, generating greater costs for public services^(10,14)^. An integrative review of 79 articles showed a decrease in studies related to the topic in the Americas and did not locate any studies published in Brazil during the period of that investigation, which showed that, in recent years, research on the topic has been developed primarily in the international context^(8)^. Furthermore, the investigation is justified by the understanding that access to and quality of emergency care are essential in the health system. Providing better working conditions in this scenario is essential for promoting worker health and improving access to these services^(15,16)^. It is important to consider the emergence of studies on CF, due to the varied manifestations of each individual who suffers from it, in addition to the diverse implications, which have a negative impact on well-being and quality of life as well as on health institutions themselves and the quality of care provided^(13)^.

Thus, the question was: what are the levels of CF in healthcare professionals working in emergency services? This topic is of interest to the team of the project “Health Work International Project (HWOPI)”, which involves researchers from two other countries and is part of the Center for Health Technology and Services Research.

## OBJECTIVES

To assess the level of CF in healthcare professionals working in emergency services and its association with socio-professional characteristics.

## METHODS

### Ethical aspects

The research followed Resolution 466/12 of the Brazilian National Health Council, which involves research with human beings. The project was assessed and approved by the *Universidade Comunitária da Região de Chapecó* Research Ethics Committee via *Plataforma Brasil*. The Informed Consent Form was obtained from all study participants in writing in two copies. After approval, the researchers underwent training to conduct the study, considering the dynamics of services and healthcare.

### Study design

This is a quantitative, descriptive and cross-sectional study. The manuscript was guided by the STrengthening the Reporting of OBservational studies in Epidemiology. The study setting consisted of services that comprise the Emergency Care Network (In Portuguese, *Rede de Atenção às Urgências e Emergências* - RUE) of the western region of Santa Catarina, southern Brazil. In Brazil, the RUE is a complex network belonging to the Brazilian Health System (In Portuguese, *Sistema Único de Saúde* - SUS). Thus, the following were included:

**Scenario I** - Mobile Emergency Care Service (In Portuguese, *Serviço de Atendimento Móvel de Urgência* - SAMU);

**Scenario II** - Emergency Care Units (ECU 24 hours) and the 24-hour Emergency Services Set, i.e., a ECU and an Emergency Care (EC);

**Scenario III** - hospital care, characterized by emergency services in two hospitals (HA), one for reference care in the SUS for adults and one for reference care for children (HC).

Data were collected by a member of the research team, using a research protocol, from July 2022 to January 2023. The research began with prior scheduling, presentation of the study, and acceptance by signing two copies of the Informed Consent Form. Participants then answered a socio-professional questionnaire containing information (age, sex, skin color, education, marital status, number of children, years of experience in the health area, time at the institution, work sector, role, specialization, weekly workload, work shift, type of shift schedule, type of employment relationship, and whether a participant works at another institution). Moreover, the self-completed Professional Quality of Life Scale (ProQOL-5)^(4)^ was used, since the research is part of the HWOPI project. The instrument consists of 30 items, subdivided into three subscales, each consisting of ten items, which assess three distinct phenomena, such as CS, BO and STS, phenomena that make up CF, assessing the negative (CF) and positive (CS) effects^(4)^. The scale has also been used because it integrates the positive component of CS, not just the negative component^(4)^. CF results from high BO and high STS.

### Population and sample

Participants who worked as physicians, nurses, nursing technicians or nursing assistants in one of the scenarios of interest and had at least three months of experience in professional practice were included. Professionals who were absent for any reason during the data collection period were excluded.

To define study participants, the eligible population was first sought, and a total of 260 professionals were identified (99 physicians, 46 nurses, 62 nursing technicians and 53 nursing assistants). A 95% confidence level and 5% sampling error were considered, with the help of SurveyMonkey (https://pt.surveymonkey.com/mp/sample-size-calculator/) and upon confirmation of the static consultancy contracted for design and analysis. Therefore, the sample was calculated at 161 participants. The entire eligible population was invited to participate in the study, and 186 professionals accepted the invitation and answered the questionnaire.

### Analysis of results, and statistics

Data were analyzed using descriptive and inferential statistics, with the help of the Statistical Package for the Social Sciences version 28. Findings were presented in absolute and relative frequencies as well as measures of central tendency, such as mean, median, maximum, minimum and standard deviation. Pearson’s correlation coefficient, parametric Student’s t-test for independent samples and nonparametric Mann-Whitney test were used. The significance limit was assumed to be p<0.05.

To calculate the ProQOL-5 cut-off points, the primary values of the subscales were converted into scores, according to the original scale^(4)^, transforming the primary values of the CS, BO and STS subscales into scores. The forced reconversion of primary values to obtain M=50 and SD=10 allows comparison between the values of the three dimensions and comparison with other studies^(17)^.

## RESULTS

A total of 186 professionals participated in the study, including 49 physicians (26.3%), 50 nurses (26.9%), 39 nursing technicians (21.0%) and 48 nursing assistants (25.8%), who were part of a young adult population, mostly female, with a partner and children. Table 1 details the profile of the RUE professionals participating in the research.

**Table 1:** Characterization of participants (N = 186), Western region, Santa Catarina, Brazil, 2023

Variables	n (%)
Age (years)	39.0^*^ ± 9.7
Sex	
Male	52 (28.0)
Female	134 (72.0)
Skin color	
Black	2 (1.1)
Brown	34 (18.3)
White	147 (79.0)
Other	3 (1.6)
Education	
High school	60 (32.3)
Higher education	50 (26.9)
Specialization	73 (39.2)
Master’s degree	3 (1.6)
Marital status	
Without a partner	68 (36.6)
With a partner	118 (63.4)
Have children	
No Yes	71 (38.2) 115 (61.8)
Number of children	1 (0 - 2)‡Mo1
Years of experience in the healthcare field	10 (5 - 20)†
Length of work at the institution (years)	4 (1 - 8)†
Work sector	
ECU	68 (36.6)
SAMU (BHU)	8 (4.3)
SAMU (ASU)	15 (8.1)
SAER	1 (0.5)
EC	44 (23.7)
Children’s Hospital ER	20 (10.8)
Adult Hospital ER	30 (16.1)
Professional category	
Nursing assistant	48 (25.8)
Nurse	50 (26.9)
Physician	49 (26.3)
Nursing technician	39 (21.0)
Weekly working hours (total hours)	40.8^*^ ± 17.6
Work shift	
Morning	42 (22.6)
Afternoon	28 (15.1)
Evening	69 (37.1)
Other §	47 (25.3)
Type of employment relationship	
Public tender/public employment	93 (50.0)
CLT	63 (33.9)
Emergency	7 (3.8)
Selection contract	23 (12.4)
Works at another institution	
No	102 (55.8)
Yes	84(45.2)
Weekly hours at another institution	34.0^*^ ± 15.9

* *Mean; Mo - mode; ±Standard deviation; †Median (P25 - P75); ‡Maximum and minimum; §11 cases in morning, afternoon and evening (5.9%), two cases in afternoon and evening (1.1%), 13 cases in morning and afternoon (7.0%), 19 cases of 12/36 shift with no defined shift (10.2%) and two did not respond (1.1%); BHU - Basic Health Unit; ECU - Emergency Care Unit; SAMU - Mobile Emergency Care Service; SAER - Air Police Service; EC - Emergency Care; ER - Emergency Room; ASU - Advanced Support Unit; CLT - Consolidation of Labor Laws.*

In Table 2, it is possible to observe the results of the application of PROQOL-5-BR.

**Table 2:** Professional Quality of Life Scale 5 (N = 186), Western region, Santa Catarina, Brazil, 2023

Subscales	Mean	Standard deviation	Low n (%)	Moderate n (%)	High n (%)
Compassion satisfaction	38.0	6.0	1 (0.5)	131 (70.4)	54 (29.0)
Burnout syndrome	19.1	5.6	133 (71.5)	53 (28.5)	-
Secondary traumatic stress	18.3	7.2	139 (74.7)	47 (25.3)	-

*Notes: - for numeric data equal to zero, not resulting from rounding.*

Table 3 shows the relationship between PROQOL-5-BR and participants’ demographic and work variables.

**Table 3:** Associations of Professional Quality of Life Scale 5 with demographic and work variables (N =186), Western region, Santa Catarina, Brazil, 2023

Variables	CS	BO	STS
Age (years)	0.204†	- 0.321‡	-0.088
Education	0.059	0.162^*^	0.146^*^
Number of children	0.228†	-0.272‡	-0.091
Years of experience in the health field	0.092	-0.227†	-0.088
Length of work at the institution (years)	0.107	-0.106	0.036
Weekly workload (total hours)	0.126	-0.061	0.016
Weekly hours in another institution	0.104	0.103	0.098

* *p<0.05; † p<0.01; ‡p<0.001; CS - compassion satisfaction; BO - burnout syndrome; STS - secondary traumatic stress.*

*Note: correlation coefficients between variables.*

The study also identified that there was a positive and statistically significant association between age and number of children and CS scores, i.e., the older the age and the greater the number of children, the higher the levels of satisfaction.

Thus, there were also significant associations related to BO, i.e., the older the person, the greater the number of children and the longer the experience in the health area, the lower the CF levels.

Finally, there was a significant negative association between participants’ level of education and the STS scores, i.e., the higher the level of education, the lower the levels of the phenomenon.

**Figure 1:**
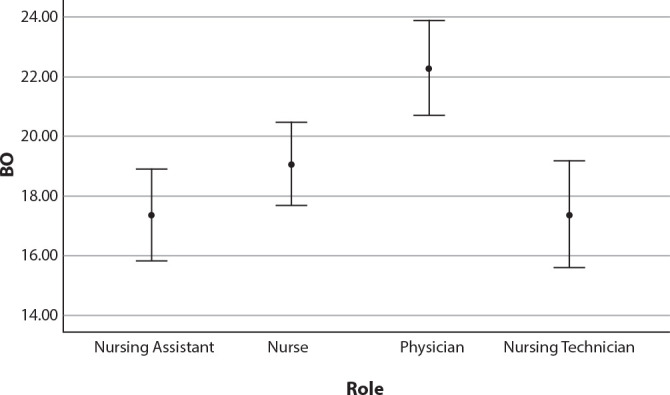
Association between role and burnout subscale (N = 186), Western region, Santa Catarina, Brazil, 2023

Physicians have significantly higher scores on the BO subscale than other professionals (p<0.001), as shown in Figure 1.

In the other subscales, there was no statistically significant difference between the roles (CS: p=0.306 and STS: p=0.087).

## DISCUSSION

In the context of emergency services, professionals need to work as a team and under pressure in different situations, including those that require rapid intervention, in a transitory context of care, sometimes permeated by intense and repetitive situations (severity of pathologies, unpredictability of situations, emotional burden and frequent physical and verbal violence); therefore, spaces that favor stress and BO^(13,18)^. Studies indicate that hospital emergency departments are a favorable setting for the development of psychosomatic illnesses^(13,19)^. Physical and psychological stress, combined with high demand and long working hours, harm quality of life, especially for emergency nurses^(13,20)^. However, it is not possible to take the elements that undermine workers’ health as a trend. Several factors related to workers’ personality and personal support, in addition to institutional aspects and work experiences, among others, interfere with how workers deal with stress, traumatic situations, CS and CF.

In this study, a significant positive association was found between age and number of children and CS scores. It is reflected that life experience potentially guides workers’ understanding of what can be changed and what is within their reach in the work context, aspects that are qualified with age. In turn, the family context with children directs workers to other demands, which potentially “disconnects” them from work. Research conducted in emergency services in Portugal supports the findings, showing that older participants have better CS rates^(13)^. Another study showed that nurses with less than five years of experience showed significantly lower CS and higher CF scores than nurses with ten or more years of experience^(20)^. Similarly, nurses with more than 20 years of experience had higher rates of CS compared to professionals with six months to two years of service^(2)^.

The relationship between age, number of children and years of experience was also statistically significant with BO scores. This research supports these findings, showing that older age correlated with lower BO and higher CS scores^(21,22)^. Another investigation showed that nurses with more than 20 years of experience had lower levels of BO compared to less experienced ones^(2)^. The set of findings allows us to reflect on accumulated expectations and experience, which are higher and lower, respectively, among professionals with less time in service, which can modulate behavior and burnout at work.

Age and experience were factors related to the increase in CS and the decrease in BO. Regarding CF, this can be better understood when thinking about compassion, since more experienced workers accumulate a diversity of experience that allows them to better distinguish which patients require greater involvement, with potential positive outcomes, and, at the same time, block those that will result in extreme distress, such as more traumatic situations. In this regard, an integrative review discussed other aspects of resilience. The development of this skill allows professionals to better deal with their work environment. This protective factor is also related to workers’ hope, optimism and self-efficacy^(12)^.

Concerning STS, there was a significant association between the level of education and the occurrence of the phenomenon. Thus, the higher the level of education, the lower the rates of this subscale. The finding suggests that higher education provides greater support for workers to face traumatic situations. This is especially important when considering that, in Brazil, there are different levels of education working in different care contexts, with an important warning for nursing assistants and technicians. In contrast, a study showed that academic level was statistically associated with BO and STS. This means that participants with a master’s/doctoral degree had mean BO grades that were 2.79 units higher than those of the others, and STS scores that were 3.14 units higher^(23)^.

Through the investigation, it was also possible to identify that physicians presented significantly higher scores on the BO subscale compared to other professionals (p<0.001). Hence, a study with 15,243 physicians working in emergency services in China showed a moderate pattern of emotional exhaustion and depersonalization, in addition to a high risk for low personal fulfillment. The data also showed that 14.9% of participants presented a high level of BO, with 46.8% with a high score for emotional exhaustion, 24% with a high score for depersonalization, and 60.5% with a high risk of low professional fulfillment^(24)^. The medical category was also significantly more affected by BO compared to others in a study involving 529 professionals from French hospitals, where almost one in two emergency room doctors had BO (50.7%)^(18)^. In contrast, a meta-analysis study, including a total sample of 79,437 participants, showed that the prevalence of BO (37.4%) was higher among nurses during the pandemic^(25)^.

Potentially, not letting oneself be overcome by emotions or being involved with patients, while maintaining distance, typical of emergency services, can serve as protection in relation to CF, but not necessarily against BO, as other research shows^(17,25)^.

Regardless of the professional category, emergency services are increasingly providing fertile ground for the occurrence of occupational syndromes and diseases, especially those related to the mental health of these workers^(18,25)^. Research has shown that 39% of frontline professionals have STS. In addition, 43% of participants had depression, including more severe depression, with 13% reporting self-harm or suicidal ideation^(25)^.

Considering that emergency services are characterized as spaces that expose workers to new potential circumstances for STS and BO, it is believed that the elements of the context studied still favor CS, protecting workers from CF. It is also reflected that the rapid outcome of cases in the emergency room, with patients being referred for discharge or other services, also limits the time spent with users, which may require less compassion from caregivers.

Research^(26)^ reveals that compassion, understood as a human and social phenomenon, contributes to the reduction of others’ pain, since it prioritizes the well-being of others, thus favoring the connection with the other person, but it brings consequences, both positive and negative, for professionals. Internal and external circumstances of individuals, such as stress and negative affect, affect CF, while positive affect and solidarity inversely influence CS. Thus, there is an oscillation between CF and CS in health work, which can be perceived as essential for human development^(26)^.

Comparing the findings with the literature reinforces that mental health associated with work depends on several factors, being an always complex relationship that requires monitoring by institutions and researchers.

The findings make us reflect on how emotional involvement with work and compassion for patients can have repercussions on the progressive loss of capacity of healthcare professionals, resulting in CF, as well as the opposite, since working in an emergency, by leaving little room for emotions, can protect professionals from CF. Thus, the need for a rapid response to patients’ condition is an aspect of this work context that potentially has repercussions on CS.

The study has limitations, potentially related to its cross-sectional nature and convenience sample, which does not allow generalizing results to other contexts. However, it can contribute to advancing research on CF in healthcare professionals, as well as bringing results to the field of occupational health, with indicators of the workers most vulnerable to CF, BO and STS.

The findings point to the need for approaches to CF, a phenomenon little addressed in Brazil, as well as the intensification of preventive measures against stress and BO, in emergency services, to promote quality of life at work and quality of care provided in these scenarios.

Evidence on CF indicates the need to make efforts to improve the work process in terms of addressing and valuing the repercussions of the phenomenon on the work process and even on the personal dimension of nurses and other health workers. From this perspective, the collaboration of managers is essential in the different care scenarios^(27)^. Studies present initiatives for preventing and coping with CF, such as the search for management strategies that aim to improve quality of life at work, continuing education practices with professionals and fostering resilience^(28-30)^.

It is also worth noting that compassion is affected by professionals’ personal experiences and characteristics associated with their capacity for altruism and empathic concern for others. Thus, professionals with these characteristics more pronounced may present a greater risk for the development of CF^(30)^.

It is also suggested that young workers with little professional experience and less education be closely monitored, with the implementation of admission programs that prepare them to face the adversities of the work context, in addition to offering psychological support to all workers. From a managerial perspective, it would be interesting to consider that the shifts bring together the most experienced and the least experienced, with positive practical implications for worker care and health.

### Study limitations

Since this is a quantitative study, understanding of the phenomenon is limited. In this sense, with the use of standardized responses, it was not possible to observe subjectivity, historical and contextual aspects, in addition to participants’ real experiences. Thus, the reasons behind the results found may remain obscure, requiring new complementary studies.

### Contributions to health, nursing, or public policy

The study provides information for health, public health, and community health as well as for occupational health nursing. Through it, it was possible to analyze, in a clearer way, the prevalence and intensity of CF among professionals working in emergency services. Furthermore, by analyzing quantitative data, it was possible to identify specific socio-occupational factors that protect against or aggravate the occurrence of the phenomenon. These results can be used to formulate occupational health policies or management strategies that meet workers’ needs, promote better working conditions and, as a consequence, higher quality in patient care. Furthermore, the study can increase the dissemination and awareness of the issue of CF, which is still little researched in Brazil.

## CONCLUSIONS

The research showed that there are moderate levels of CS and low levels of BO and STS among participants. In the sample, variables such as age and number of children define the CS and BO scores, being positive for both, i.e., they favor the first and disfavor the second. Furthermore, the longer the professional experience in health, the lower the scores on the BO subscale, with the medical category showing significantly higher scores on this subscale than the other professionals.

In turn, the level of education proved to be the predictive variable for STS, signaling the profile of professionals less susceptible to CF in the set of findings, which is essential for defining measures to prevent phenomena that result in worker illness.

It is concluded that individual characteristics (age and having children) and work/training characteristics (length of experience in the health area and level of education) reflect exposure to CF. The research shows the importance of implementing managerial and institutional strategies that promote well-being at work and stress management, considering worker characteristics. Thus, actions need to be considered individually and collectively, with benefits for both the caregiver and the person being cared for.
