# Supplementary material for: Compassion fatigue in emergency care professionals
Source: Rev Bras Enferm. 2025 Jun 20;78(2):e20230367. doi: 10.1590/0034-7167-2023-0367 (PMC12185142; doi:10.1590/0034-7167-2023-0367)
Supplement: Translation

# Translation: Fadiga por compaixão em profissionais de serviços de urgência e emergência

**DOI:** 10.1590/0034-7167-2023-0367pt

**Authors:** Letícia de Lima Trindade, Maiara Daís Schoeninger, Josiane Carneiro, Elisabete Maria das Neves Borges, Clarissa Bohrer da Silva, Carine Vendruscolo, Fernanda Karla Metelski

**Affiliations:** I Universidade do Estado de Santa Catarina. Chapecó, Santa Catarina, Brasil; II Universidade Comunitária da região de Chapecó. Chapecó, Santa Catarina, Brasil; III Escola Superior de Enfermagem do Porto. Porto, Portugal

**Keywords:** Fadiga por Compaixão, Serviços Médicos de Emergência, Pessoal de Saúde, Saúde Ocupacional, Riscos Ocupacionais.

## Abstract

**Objetivos:**

avaliar o nível de fadiga por compaixão em profissionais de saúde que atuam em serviços de urgência e emergência e sua associação com as características socioprofissionais.

**Métodos:**

pesquisa transversal, realizada nos anos de 2022 e 2023, com 186 trabalhadores de saúde. Utilizaram-se questionário sociolaboral e a *Professional Quality of Life Scale* 5.

**Resultados:**

evidenciaram-se maiores médias de satisfação por compaixão, seguidas de síndrome de *burnout* e Estresse Traumático Secundário. Idade e número de filhos foram relacionados à satisfação por compaixão. Estas variáveis e os anos de experiência na área da saúde foram inversamente correlacionados com os escores de *burnout*. Ainda, quanto maior o nível de escolaridade, menores os escores para o Estresse Traumático Secundário.

**Conclusões:**

a satisfação por compaixão exerce proteção para a maioria dos participantes do estudo, sendo importante investir nela para evitar fadiga por compaixão nesse cenário.

## INTRODUÇÃO

Os serviços de saúde são considerados locais que exponenciam diversas ameaças à saúde dos trabalhadores. Entre essas ameaças, destaca-se a exposição aos riscos psicossociais relacionados à concepção, organização e gestão do trabalho^(1)^. A complexidade da área da saúde e os desafios de prestar assistência de qualidade colocam o profissional em um nível elevado de estresse, o que favorece, entre outros agravos, a fadiga por compaixão (FC)^(2)^. Essa fadiga se caracteriza por comportamentos e emoções resultantes do processo de cuidado ou de querer ajudar uma pessoa traumatizada ou em sofrimento, o que leva aquele que cuida/ajuda a um estado de exaustão biológica, psicológica e social^(3)^. Na sua origem teórica, a FC tem por base o modelo de qualidade de vida profissional (QVP), o qual a apresenta como resultante da combinação de síndrome de *burnout* (BO), Estresse Traumático Secundário (ETS) e reduzida satisfação por compaixão (SC)^(3)^. Neste sentido, é válido ressaltar que o BO e o ETS não são sinônimos da FC, mas o somatório deles influencia o seu desenvolvimento.

A QVP pode ser entendida como a qualidade que o profissional sente em relação ao seu trabalho^(3,4)^. O modelo incorpora dois aspectos: o positivo (SC) e o negativo (FC). O primeiro refere-se ao prazer que o trabalhador sente ao realizar bem o seu trabalho, estando relacionado ao fato de poder ajudar os outros, ao sentimento positivo em relação aos seus colegas e à capacidade de contribuir para o ambiente de trabalho ou até mesmo para o bem maior da sociedade. Por outro lado, o segundo divide-se em duas dimensões: BO, processo de estresse laboral crônico, que envolve a tríade exaustão emocional, despersonalização e baixa realização pessoal; e ETS, motivado pelo medo e trauma relacionado ao trabalho^(4)^.

Entre esses aspectos, o BO pode ser definido como fenômeno ocupacional causado por altos níveis de estresse, vivenciados em longo prazo e administrados sem sucesso, causando o estado de exaustão emocional, despersonalização e sentimentos de incompetência^(5)^. Por sua vez, o ETS é caracterizado como uma síndrome ocupacional oriunda do estresse, causado pela exposição a incidentes vivenciados por outra pessoa^(3)^. A SC é conceituada como a gratificação proporcionada pelo ato de cuidar de pessoas em situações de sofrimento. Quando os profissionais atuantes nos serviços de saúde se sentem valorizados e estão satisfeitos com suas relações interpessoais e laborais, a SC atua como um fator protetor da FC^(3,6)^.

Estudo mostrou índices elevados de FC, que afetam 52,55% dos profissionais atuantes nos serviços de saúde. Além disso, o BO totalizou 51,98%, e a SC, 47,55%, respectivamente^(7)^. Revisão integrativa, incluindo mais de 28 mil enfermeiros de 11 países, mostrou que os níveis do fenômeno aumentaram de forma gradual nos últimos dez anos, o que acende um alerta para a gravidade do problema entre profissionais de saúde, bem como a necessidade de estratégias e intervenções que proporcionem melhorias no setor^(8)^. Segunda revisão sistemática, que incluiu 71 artigos, mostrou que o fenômeno existe em diversos grupos e especialidades, e pode ser medido com sucesso ao se utilizar a ferramenta *Professional Quality of Life* (ProQOL)^(9)^.

Entre as consequências mais citadas na literatura, a FC causa redução da capacidade e interesse dos profissionais em atuar de modo empático diante de situações de sofrimento alheio, sendo considerado este o “custo de cuidar”^(10)^, podendo impactar o desempenho profissional e colocar em risco a segurança do paciente^(11)^. Nesta direção, estudos sobre FC têm contribuições para a saúde do trabalhador, para a promoção da cultura de segurança nas instituições, bem como para a cultura de paz.

Ainda, as consequências deixadas pela pandemia causada pela doença por coronavírus (COVID-19) intensificaram os fatores estressores, os quais impactaram diretamente a saúde física e mental dos trabalhadores^(11,12)^. À medida que esses profissionais experienciam um novo conjunto de circunstâncias causadas pelo ETS, a ocorrência da FC aumenta e a satisfação no trabalho diminui^(11)^.

Entre os profissionais mais afetados, destacam-se os atuantes nos serviços de urgência e emergência, que diariamente deparam-se com situações clínicas estressantes, incluindo eventos de violência, acidentes, traumas e mortes^(13)^. Além disso, são esses mesmos profissionais que, por vezes, precisam lidar e transmitir notícias ruins aos acompanhantes e familiares, gerando um novo fator de estresse^(13)^, colocando-os vulneráveis à FC.

Cabe destacar que a FC é um fenômeno que requer mais investigações, e pode ser considerado uma das principais ameaças à saúde mental dos profissionais. Tem impacto na qualidade dos cuidados prestados, gerando maiores gastos para os serviços públicos^(10,14)^. Revisão integrativa com 79 artigos mostrou diminuição de estudos relacionados à temática nas Américas e não localizou estudos publicados no Brasil no período daquela investigação, o que evidenciou que, nos últimos anos, as pesquisas no tema se desenvolveram prioritariamente no contexto internacional^(8)^. Ainda, justifica-se a investigação por compreender que o acesso e a qualidade do atendimento de emergência são essenciais no sistema de saúde. Proporcionar melhores condições de trabalho nesse cenário é fundamental para a promoção da saúde dos trabalhadores e qualificação do acesso nesses serviços^(15,16)^. É relevante considerar a emergência de estudos sobre FC, devido às manifestações variadas de cada indivíduo que sofre com ela, além das implicações diversas, o que se repercute negativamente no bem-estar e na qualidade de vida, bem como nas próprias instituições de saúde e na qualidade dos cuidados prestados^(13)^.

Nessa direção, questionou-se: quais os níveis de FC em profissionais de saúde que atuam em serviços de urgência e emergência? A temática é de interesse da equipe do projeto “*Health Work International Project* (HWOPI)”, que envolve pesquisadores de outros dois países e integra o *Center for Health Technology and Services Research*.

## OBJETIVOS

Avaliar o nível de FC em profissionais de saúde que atuam em serviços de urgência e emergência e sua associação com as características socioprofissionais.

## MÉTODOS

### Aspectos éticos

A pesquisa seguiu a Resolução nº 466/12 do Conselho Nacional de Saúde, que envolve pesquisas com seres humanos. O projeto foi apreciado e aprovado pela Comissão de Ética em Pesquisa da Universidade Comunitária da Região de Chapecó, via Plataforma Brasil. O Termo de Consentimento Livre e Esclarecido foi obtido de todos os participantes do estudo por meio escrito em duas vias. Após a aprovação, os pesquisadores passaram por treinamento para condução do estudo, considerando a dinâmica dos serviços e os cuidados sanitários.

### Desenho do estudo

Estudo quantitativo, descritivo e transversal. O manuscrito foi orientado pelo instrumento *STrengthening the Reporting of OBservational studies in Epidemiology*. O cenário de estudo foi composto por serviços que contemplam a Rede de Atenção às Urgências e Emergências (RUE) da região Oeste de Santa Catarina, no Sul do Brasil. No Brasil, a RUE é uma rede complexa pertencente ao Sistema Único de Saúde (SUS). Assim, incluíram-se:

**Cenário I** - Serviço de Atendimento Móvel de Urgência (SAMU);

**Cenário II** - Unidades de Pronto Atendimento (UPA 24 horas) e o Conjunto de Serviços de Urgência 24 horas, ou seja, uma UPA e um Pronto Atendimento (PA);

**Cenário III** - atenção hospitalar, caracterizado pelos serviços de urgência e emergência de dois hospitais (HA), um para atendimento de referência no SUS aos adultos e um de referência do público infantil (HC).

Os dados foram coletados por membro da equipe de pesquisadores, mediante protocolo de condução da pesquisa, no período de julho de 2022 a janeiro de 2023. A pesquisa iniciou-se com agendamento prévio, apresentação do estudo e aceitação mediante assinatura do Termo de Consentimento Livre e Esclarecido em duas vias. Na sequência, os participantes responderam a um questionário socioprofissional contendo informações (idade, sexo, cor da pele, escolaridade, situação conjugal, quantitativo de filhos, anos de experiência na área da saúde, tempo na instituição, setor de trabalho, função, especialização, carga horária semanal, turno de trabalho, tipo de escala de plantão, tipo de vínculo empregatício e se atua em outra instituição). Ainda, utilizou-se a *Professional Quality of Life Scale* (ProQOL-5)^(4)^ autopreenchida, pelo fato de que a pesquisa integra o projeto HWOPI. O instrumento é constituído por 30 itens, subdivididos em três subescalas, cada uma delas formada por dez itens, que avaliam três fenômenos distintos, como SC, BO e ETS, fenômenos estes que compõem a FC, avaliando os efeitos negativos (FC) e positivos (SC)^(4)^. A escala tem sido utilizada também porque integra o componente positivo de SC, não apenas o componente negativo^(4)^. A FC resulta de elevado BO e elevado ETS.

### População e amostra

Foram incluídos participantes que atuam como médico, enfermeiro, técnico ou auxiliar de enfermagem em um dos cenários de interesse com pelo menos três meses de experiência no exercício profissional. Foram excluídos os profissionais afastados por qualquer motivo no período da coleta de dados.

Para a definição dos participantes do estudo, primeiramente, buscou-se a população elegível, sendo identificado um total de 260 profissionais (sendo 99 médicos, 46 enfermeiros, 62 técnicos de enfermagem e 53 auxiliares de enfermagem). Foram considerados 95% de confiança e 5% de erro amostral, com auxílio do *SurveyMonkey* (https://pt.surveymonkey.com/mp/sample-size-calculator/) e mediante confirmação de assessoria estática contratada para desenho e análises. Sendo assim, a amostra foi calculada em 161 participantes. Toda a população elegível foi convidada a participar do estudo, sendo que 186 profissionais aceitaram o convite e responderam ao questionário.

### Análise dos resultados e estatística

Os dados foram analisados por meio da estatística descritiva e inferencial, com auxílio do *Statistical Package for the Social Sciences*, versão 28. Os achados foram apresentados em frequências absolutas e relativas, além de medidas de tendência central, como média, mediana, máximo, mínimo e desvio padrão. Foram utilizados o coeficiente de correlação de Pearson, o teste paramétrico t de *Student* para amostras independentes e o teste não paramétrico de Mann-Whitney. Assumiu-se o limite de significância de p<0,05.

Para o cálculo dos pontos de corte da ProQOL-5, transformaram-se os valores primários das subescalas em escores, conforme a escala original^(4)^, transformando os valores primários das subescalas SC, BO e ETS em escores. A reconversão forçada dos valores primários para obter M=50 e DP=10 possibilita a comparação entre os valores das três dimensões e a comparação com outros estudos^(17)^.

## RESULTADOS

Participaram do estudo 186 profissionais, sendo 49 médicos (26,3%), 50 enfermeiros (26,9%), 39 técnicos de enfermagem (21,0%) e 48 auxiliares de enfermagem (25,8%), os quais integram uma população adulta jovem, majoritariamente do sexo feminino, com companheiro e filhos. A Tabela 1 detalha o perfil dos profissionais da RUE participantes da pesquisa.

**Tabela 1:** Caracterização dos participantes (N = 186), Região Oeste, Santa Catarina, Brasil, 2023

Variáveis	n (%)
Idade (anos)	39,0^*^ ± 9,7
Sexo	
Masculino	52 (28,0)
Feminino	134 (72,0)
Cor da pele	
Negra	2 (1,1)
Parda	34 (18,3)
Branca	147 (79,0)
Outra	3 (1,6)
Escolaridade	
Ensino médio	60 (32,3)
Ensino superior	50 (26,9)
Especialização	73 (39,2)
Mestrado	3 (1,6)
Situação conjugal	
Sem companheiro	68 (36,6)
Com companheiro	118 (63,4)
Possui filhos	
Não Sim	71 (38,2) 115 (61,8)
Número de filhos	1 (0 - 2)‡Mo1
Anos de experiência na área da saúde	10 (5 - 20)†
Tempo na instituição (anos)	4 (1 - 8)†
Setor de trabalho	
UPA	68 (36,6)
SAMU (UBS)	8 (4,3)
SAMU (USA)	15 (8,1)
SAER	1 (0,5)
PA	44 (23,7)
PS Hospital Infantil	20 (10,8)
PS Hospital Adulto	30 (16,1)
Categoria profissional	
Auxiliar de enfermagem	48 (25,8)
Enfermeiro	50 (26,9)
Médico	49 (26,3)
Técnico em enfermagem	39 (21,0)
Carga horária semanal (horas totais)	40,8^*^ ± 17,6
Turno de trabalho	
Manhã	42 (22,6)
Tarde	28 (15,1)
Noite	69 (37,1)
Outro §	47 (25,3)
Tipo de vínculo empregatício	
Concurso público/Emprego público	93 (50,0)
CLT	63 (33,9)
Emergencial	7 (3,8)
Contrato seletivo	23 (12,4)
Trabalha em outra instituição	
Não	102 (55,8)
Sim	84(45,2)
Horas semanais em outra instituição	34,0^*^ ± 15,9

* *Média; Mo - moda; ±Desvio padrão; †Mediana (P25 - P75); ‡Máxima e mínima; §11 casos de manhã, tarde e noite (5,9%), dois casos de tarde e noite (1,1%), 13 casos de manhã e tarde (7,0%), 19 casos de escala 12/36 sem turno definido (10,2%) e dois não responderam (1,1%); UBS - Unidade Básica de Saúde; UPA - Unidade de Pronto Atendimento; SAMU - Serviço de Atendimento Móvel de Urgência; SAER - Serviço Aeropolicial; PA- Pronto Atendimento; PS - Pronto-Socorro; USA - Unidade de Suporte Avançado; CLT - Consolidação das Leis do Trabalho.*

Na Tabela 2, é possível observar os resultados da aplicação do PROQOL-5-BR.

**Tabela 2:** *Professional Quality of Life Scale 5* (N = 186), Região Oeste, Santa Catarina, Brasil, 2023

Subescalas	Média	Desvio padrão	Baixa n (%)	Moderada n (%)	Alta n (%)
Satisfação por compaixão	38,0	6,0	1 (0,5)	131 (70,4)	54 (29,0)
Síndrome de *burnout*	19,1	5,6	133 (71,5)	53 (28,5)	-
Estresse Traumático Secundário	18,3	7,2	139 (74,7)	47 (25,3)	-

*Notas: - para dado numérico igual a zero, não resultante de arredondamento.*

Na Tabela 3, observa-se a relação entre a PROQOL-5-BR e as variáveis demográficas e laborais dos participantes.

**Tabela 3:** Associações da *Professional Quality of Life Scale 5* com variáveis demográficas e laborais (N = 186), Região Oeste, Santa Catarina, Brasil, 2023

Variáveis	SC	BO	ETS
Idade (anos)	0,204†	- 0,321‡	-0,088
Escolaridade	0,059	0,162^*^	0,146^*^
Número de filhos	0,228†	-0,272‡	-0,091
Anos de experiência na área da saúde	0,092	-0,227†	-0,088
Tempo na instituição (anos)	0,107	-0,106	0,036
Carga horária semanal (horas totais)	0,126	-0,061	0,016
Horas semanais em outra instituição	0,104	0,103	0,098

* *p<0,05; † p<0,01; ‡p<0,001; SC - satisfação por compaixão; BO - síndrome de burnout; ETS - Estresse Traumático Secundário.*

*Nota: coeficientes de correlação entre as variáveis.*

O estudo identificou ainda que houve associação positiva e estatisticamente significativa entre idade e número de filhos e os escores de SC, ou seja, quanto maior a idade e maior o número de filhos, maiores os níveis de satisfação.

Neste sentido, também houve associações significativas relacionadas ao BO, ou seja, quanto maior a idade, maior o número de filhos e mais tempo de experiência na área da saúde, menores os níveis de FC.

Por último, houve associação negativa significativa entre o nível de escolaridade dos participantes e os escores de ETS, ou seja, quanto maior a escolaridade, menores os níveis do fenômeno.

Os médicos apresentam escores significativamente maiores da subescala de BO do que os demais profissionais (p<0,001), conforme apresenta a Figura 1.

**Figura 1:** Associação entre a função e a subescala de *burnout* (N = 186). Região Oeste, Santa Catarina, Brasil, 2023

Nas demais subescalas, não houve diferença estatisticamente significativa entre as funções (SC: p=0,306 e ETS: p=0,087).

## DISCUSSÃO

No contexto dos serviços de urgência e emergência, os profissionais precisam atuar em equipe e sob pressão em situações diversas, entre elas aquelas que requerem rápida intervenção, em um contexto transitório de cuidados, por vezes permeado por situações intensas e repetitivas próprias (gravidade das patologias, imprevisibilidade das situações, carga emocional e frequente violência física e verbal); logo, espaços que favorecem o estresse e BO^(13,18)^. Estudos indiciam o setor de urgência de hospitais como cenário propício para o desenvolvimento de doenças psicossomáticas^(13,19)^. As tensões físicas e psicológicas, aliadas à grande demanda e elevada carga horária de trabalho, prejudicam a qualidade de vida, especialmente do enfermeiro na emergência^(13,20)^. Contudo, não é possível tomar como tendência os elementos que desgastam a saúde dos trabalhadores. Diversos fatores da personalidade e suporte pessoal do trabalhador, além de aspectos institucionais e vivências laborais, entre outros, interferem em como o trabalho enfrenta o estresse, as situações traumáticas, a SC e a FC.

No presente estudo, evidenciou-se associação positiva significativa entre idade e número de filhos e os escores de SC. Reflete-se que, potencialmente, a experiência de vida direciona a compreensão do trabalhador acerca do que pode ser mudado e que está dentro do seu alcance no contexto laboral, aspectos que se qualificam com a idade. Por sua vez, o contexto familiar com filhos direciona o trabalhador para outras demandas, o que potencialmente o “desliga” do trabalho. Pesquisa realizada nos serviços de urgência e emergência de Portugal corrobora os achados, evidenciando que os participantes com maior idade apresentam melhores índices de SC^(13)^. Outra pesquisa mostrou que enfermeiros com menos de cinco anos de experiência mostraram significativamente menor SC e maiores índices de FC do que enfermeiros com dez ou mais anos de experiência^(20)^. Do mesmo modo, enfermeiros com mais de 20 anos de experiência tiveram maiores índices de SC em comparação aos profissionais com seis meses a dois anos de serviço^(2)^.

A relação entre idade, número de filhos e anos de experiência também foi estatisticamente significativa com os escores de BO. Esta pesquisa corrobora esses achados, mostrando que a idade mais avançada se correlacionou com pontuações mais baixas de BO e mais altas de SC^(21,22)^. Outra investigação mostrou que enfermeiros com mais de 20 anos de experiência apresentaram níveis mais baixos de BO em comparação aos menos experientes^(2)^. O conjunto dos achados permite refletir sobre a expectativa e axperiência acumuladas, que são maiores e menores, respectivamente, entre os profissionais com menos tempo de serviço, o que pode ser modulador de condutas e do desgaste no trabalho.

A idade e o tempo de experiência foram fatores relacionados ao aumento da SC e à diminuição do BO. No que se refere à FC, isso pode ser mais bem compreendido ao pensar acerca da compaixão, uma vez que trabalhadores mais experientes acumulam uma diversidade de experiência que permite distinguir melhor quais pacientes exigem um envolvimento maior, com potenciais desfechos positivos, e, ao mesmo tempo, bloquear aqueles que repercutirão em sofrimento extremo, a exemplo das situações mais traumáticas. Nessa direção, revisão integrativa discutiu outros aspectos da resiliência. O desenvolvimento dessa habilidade permite que profissionais consigam lidar melhor com o seu ambiente de trabalho. Esse fator de proteção também está relacionado à esperança, ao otimismo e à autoeficácia dos trabalhadores^(12)^.

Em relação ao ETS, houve uma associação significativa entre o nível de escolaridade e ocorrência do fenômeno. Desse modo, quanto maior a escolaridade, menores os índices dessa subescala. O achado sugere que formação em nível superior dá maior suporte para o trabalhador enfrentar as situações traumáticas. Isso é especialmente importante ao considerar que, no Brasil, há diferentes níveis formativos atuando nos diferentes contextos assistenciais, com alerta importante para os auxiliares e técnicos de enfermagem. Em contrapartida, estudo evidenciou que o nível acadêmico foi estatisticamente associado ao BO e ETS. Isso significa que os participantes com mestrado/doutorado apresentavam graus médios de BO, que foram 2,79 unidades mais altos do que os dos demais, e pontuações de ETS, que foram 3,14 unidades mais altas^(23)^.

Por meio da investigação, foi possível identificar ainda que médicos apresentaram escores significativamente maiores da subescala de BO em comparação aos demais profissionais (p<0,001). Nesse sentido, pesquisa com 15.243 médicos atuantes nos serviços de urgência e emergência da China mostrou um padrão moderado de exaustão emocional e despersonalização, além do elevado risco para baixa realização pessoal. Os dados mostraram ainda que 14,9% dos participantes apresentaram alto nível de BO, sendo 46,8% com pontuação alta para exaustão emocional, 24%, com pontuação elevada para despersonalização, e 60,5%, com alto risco de baixa realização profissional^(24)^. A categoria médica também foi significativamente mais afetada pelo BO em comparação aos demais em estudo envolvendo 529 profissionais de hospitais franceses, onde quase um em cada dois médicos do pronto-socorro teve BO (50,7%)^(18)^. Diferente disso, estudo de meta-análise, incluindo uma amostra total de 79.437 participantes, evidenciou que a prevalência de BO (37,4%) foi maior entre enfermeiros durante a pandemia^(25)^.

Potencialmente, não se deixar invadir por emoções ou envolver-se com o paciente, com manutenção do distanciamento, típico dos serviços de urgência, pode servir como proteção em relação à FC, mas não necessariamente ao BO, como mostram outras investigações^(17,25)^.

Independentemente da categoria profissional, os serviços de urgência e emergência estão cada vez mais proporcionando terreno fértil para a ocorrência de síndromes e doenças ocupacionais, principalmente as relacionadas à saúde mental desses trabalhadores^(18,25)^. Investigação evidenciou índices de 39% de ETS em profissionais atuantes na linha de frente. Além disso, 43% dos participantes apresentaram depressão, inclusive de forma mais grave, já que 13% deles relataram automutilação ou ideação suicida^(25)^.

Levando em consideração que os serviços de urgência e emergência se tipificam como espaços que expõem os trabalhadores a novas circunstâncias potenciais para ETS e BO, acredita-se que os elementos do contexto pesquisado ainda favorecem a SC, protegendo o trabalhador da FC. Reflete-se também que o desfecho rápido dos casos na urgência, com encaminhamento do paciente para alta ou outros serviços, também limita o tempo de convivência com o usuário, o que pode exigir menor compaixão daquele que cuida.

Investigação^(26)^ revela que a compaixão, compreendida como um fenômeno humano e social, contribui para a diminuição da dor alheia, uma vez que prioriza o bem-estar do outro, favorecendo, assim, a conexão com a outra pessoa, porém traz consequências, tanto positivas quanto negativas, para o profissional. Circunstâncias internas e externas do indivíduo, como estresse e afeto negativo, afetam a FC, enquanto afeto positivo e solidariedade influenciam de modo inverso a SC. Assim, ocorre uma oscilação entre FC e SC no trabalho em saúde, o qual pode ser percebido como essencial para o desenvolvimento humano^(26)^.

O confronto dos achados com a literatura reforça que a saúde mental associada ao trabalho depende de diversos fatores, sendo uma relação sempre complexa que requer acompanhamento das instituições e dos pesquisadores.

Os achados fazem refletir sobre como o envolvimento emocional com o trabalho e compaixão com o paciente podem repercutir na perda progressiva da capacidade do profissional de saúde, resultando em FC, bem como o contrário, pois a atuação em emergência, por deixar pouco espaço para emoções, pode proteger os profissionais da FC. Assim, a necessidade de resposta rápida à condição do doente é um aspecto desse contexto de trabalho que potencialmente repercute em SC.

O estudo apresenta limitações, potencialmente relacionadas ao seu caráter transversal e à amostra de conveniência, o que não permite generalizar resultados para outros contextos. Contudo, pode contribuir para avançar com investigações acerca da FC em profissionais de saúde, bem como trazer resultados para o campo da saúde do trabalhador, com indicativos dos trabalhadores mais vulneráveis à FC, BO e ETS.

Os achados apontam para a necessidade de abordagens da FC, fenômeno pouco abordado no Brasil, bem como a intensificação de medidas preventivas ao estresse e BO, nos serviços de emergência, para promover a qualidade de vida no trabalho e qualidade da assistência prestada nesses cenários.

As evidências sobre FC sinalizam para a necessidade de depositar esforços, a fim de favorecer a melhoria do processo de trabalho no que tange ao enfrentamento e valorização das repercussões do fenômeno no processo de trabalho e até mesmo na dimensão pessoal dos enfermeiros e demais trabalhadores de saúde. Nessa perspectiva, a colaboração dos gestores é fundamental nos diferentes cenários da assistência^(27)^. Estudos apresentam iniciativas de prevenção e enfrentamento da FC, como a busca por estratégias gerenciais que visem melhorias da qualidade de vida no trabalho, práticas de educação continuada com os profissionais e fomento à resiliência^(28-30)^.

Cabe ressaltar também que a compaixão é afetada pelas vivências pessoais e características do profissional, associada à sua capacidade de altruísmo e preocupação empática com os demais. Assim, profissionais com essas características mais acentuadas podem apresentar maior risco para o desenvolvimento da FC^(30)^.

Sugere-se ainda um olhar atento aos trabalhadores jovens, com pouca experiência profissional e com menor escolaridade, com a implantação de programas admissionais que os preparem para o enfrentamento das adversidades do contexto de trabalho, além da oferta de suporte psicológico para todos os trabalhadores. Gerencialmente, seria interessante considerar que as escalas reunissem os mais experientes com os menos experientes, com implicações práticas positivas para a assistência e para a saúde do trabalhador.

### Limitações do estudo

Por se tratar de estudo quantitativo, a compreensão do fenômeno é limitada. Nesse sentido, com a utilização de respostas padronizadas, não foi possível observar subjetividade, aspectos históricos e contextuais, além das experiências reais dos participantes. Desse modo, as razões por trás dos resultados encontrados podem permanecer obscurecidas, necessitando de novos estudos complementares.

### Contribuições para as áreas da saúde, enfermagem, ou políticas públicas

O estudo oferece informações para as áreas da saúde, saúde pública e saúde comunitária, bem como para a enfermagem do trabalho. Por meio dele, foi possível analisar, de modo mais claro, a prevalência e intensidade da FC entre os profissionais atuantes nos serviços de urgência e emergência. Além disso, ao analisar os dados quantitativos, foi possível identificar fatores sociolaborais específicos que protegem ou agravam a ocorrência do fenômeno. Esses resultados podem ser utilizados para a formulação de políticas de saúde ocupacional, ou estratégias gerenciais, que atendam às necessidades dos trabalhadores, promovam melhores condições de trabalho e, como consequência, maior qualidade no atendimento prestado ao paciente. Ainda, o estudo pode ampliar a divulgação e sensibilização quanto à temática da FC, que ainda é pouco investigada no Brasil.

## CONCLUSÕES

A investigação permitiu verificar que existem níveis moderados de SC e baixo de BO e ETS entre os participantes. Na amostra, variáveis como idade e número de filhos são definidoras dos escores de SC e BO, sendo positivos para ambos, ou seja, favorecem a primeira e desfavorecem o segundo. Ainda, quanto maior o tempo de experiência profissional na área da saúde, menores os escores da subescala de BO, sendo que a categoria médica mostrou escores significativamente maiores nessa subescala do que os demais profissionais.

Por sua vez, o nível de escolaridade mostrou-se como a variável preditiva para o ETS, sinalizando o perfil dos profissionais menos suscetíveis à FC no conjunto dos achados, o que é fundamental para definir medidas de prevenção dos fenômenos que resultam no adoecimento do trabalhador.

Conclui-se que características do indivíduo (idade e possuir filhos) e laborais/formação (tempo de experiência na área da saúde e nível de escolaridade) refletem a exposição à FC. A pesquisa mostra a importância da inserção de estratégias gerenciais e institucionais promotoras de bem-estar no trabalho e de gerenciamento do estresse, considerando as características do trabalhador. Assim, as ações precisam ser pensadas de modo individual e coletivo, com benefícios para quem cuida e para quem é cuidado.
